# Dynamic of the somatosensory system in postherpetic neuralgia

**DOI:** 10.1097/PR9.0000000000000668

**Published:** 2018-10-26

**Authors:** Janne Gierthmühlen, Olga Braig, Stefanie Rehm, Jana Hellriegel, Andreas Binder, Ralf Baron

**Affiliations:** Division of Neurological Pain Research and Therapy, Department of Neurology, University Hospital of Schleswig-Holstein, Kiel, Germany

**Keywords:** QST, Postherpetic neuralgia, Chronic pain, Somatosensory profiles, Mechanism-based treatment

## Abstract

**Introduction:**

In postherpetic neuralgia (PHN) different types of patients can be distinguished regarding their predominant peripheral nociceptor function.

**Objective:**

The aim was to examine somatosensory profiles in the course of disease with special regard to the different subtypes existing in PHN.

**Methods:**

Twenty patients with PHN (7 men and 13 women, age 67 ± 9.6 years) were examined at baseline (disease duration 18.1 ± 26 months) and follow-up (31.6 ± 23.8 months later) with quantitative sensory testing (protocol of the German Research Network on Neuropathic Pain).

**Results:**

Fourteen (70%) PHN patients presented with impaired (iPHN) and 6 (30%) with preserved (pPHN) C-fiber function. Groups did not differ regarding age, disease duration, or pain intensity at baseline. Both groups did not differ regarding change in pain intensity (−0.5 ± 2.3 vs −1.7 ± 2.6 numerical rating scale, *P* = n.s.) at follow-up. Impaired PHN improved in thermal and mechanical detection thresholds as well as allodynia independent from change in pain intensity. By contrast, pPHN showed an increase in mechanical pain sensitivity (1.4 ± 2.5 vs −0.4 ± 2.2, *P* < 0.05) and a trend towards a stronger loss of detection (66% vs 33%, *P* = n.s.) on follow-up.

**Conclusion:**

Results demonstrate that patients with preserved C-fiber function are more predisposed to develop signs of central sensitization as demonstrated by an increased mechanical pain sensitivity. Impaired C-fiber function is able to improve even in chronic cases, but a functional loss is unlikely to play a role here. The knowledge of development of somatosensory profiles in the course of the disease offers possibilities to optimize a mechanism-based treatment.

## 1. Introduction

Postherpetic neuralgia (PHN) is the most common complication of herpes zoster infection.^14^ It is often characterized by the presence of spontaneous pain, loss of thermal and mechanical detection, as well as an exaggerated pain response to noxious (hyperalgesia) and non-noxious (allodynia) stimuli.^15^ Different types of patients can be distinguished regarding their predominant cutaneous nociceptor function^7^: patients who primarily present with sensory loss, ie, demonstrate cutaneous nociceptor deafferentation and those who do not show sensory loss, ie, have preserved or even sensitized cutaneous nociceptors.^16^ In patients with sensory loss increased spontaneous activity in deafferented central neurons and/or reorganization of central connections might play a role in pain generation. Pain and somatosensory abnormalities of PHN patients with preserved or even sensitized nociceptors might be due to an abnormal sensitization of unmyelinated cutaneous nociceptors (irritable nociceptors^7^). To date, it is unclear why some patients have the impaired and others the preserved clinical phenotype. However, the assessment of these specific sensory symptoms and signs enables to identify the underlying mechanism that can help to understand pathophysiology and improve treatment in the concept of a mechanism-based therapy.^28^ Because it has been shown that a specific symptom may be generated by several different mechanisms, it became clear that a specific somatosensory constellation might be more suitable to mirror the mechanisms instead of a single sign.^1,16^ Standardized quantitative sensory testing (QST) enables to investigate different afferent nerve fiber functions or their central pathways and create an individual somatosensory profile.^9^ The presence of different somatosensory signs and their constellation are suggested to be linked to underlying mechanisms.^1^

Several studies have investigated somatosensory function of patients with PHN^21,26,31^; however, investigations regarding changes of somatosensory signs and symptoms in the course of PHN are rare. The aim of our study was therefore to examine somatosensory signs and symptoms in the course of disease with special regard to the different subtypes existing in PHN.

## 2. Methods

### 2.1. Experimental set-up

Twenty patients with PHN (7 men and 13 women, age 67 ± 9.6 years, range 40–77 years) were examined at baseline (disease duration 18.1 ± 26, range 1–97 months) and follow-up visit (31.6 ± 23.8 months later). Recruitment consisted of all patients with PHN who had been included into the database of the German Research Network on Neuropathic Pain in Kiel, Germany, between 2004 and 2007 who agreed to participate in a follow-up examination. Postherpetic neuralgia was present in trigeminal innervation territory (n = 5), cervical (n = 1), thoracic (n = 11), as well as lumbar (n = 3) dermatomes. The initial visit's (baseline) data set was provided by the database of the German Research Network on Neuropathic Pain. The examination of both baseline and follow-up visit followed an identical algorithm and included medical history (pain intensity, disease duration, and treatment) and QST (protocol of the German Research Network on Neuropathic Pain^9^) on the affected and corresponding contralateral body side. Patients with any neurological comorbidity that could otherwise influence testing results such as polyneuropathy, diabetes, vascular disease, etc., as well as patients with skin lesions or dermatological disorders in the areas to be tested or with difficulties in German language skills were excluded from the study. The study was in accordance with the Declaration of Helsinki and approved by the institutional review board of the Faculty of Medicine at Christian-Albrechts-University of Kiel. All patients gave written informed consent to take part in the study.

### 2.2. Assessment of sensory signs and symptoms

Current and mean pain intensity during the week before examination was measured with a numerical rating scale (NRS, where 0 = no pain and 10 = maximum pain imaginable). For assessment of somatosensory function, the QST protocol of the German Research Network on Neuropathic Pain was used.^29,30^ This protocol contains the investigation of 13 different parameters that provide a complete somatosensory profile of a person.^29^ It includes the investigation of mechanical detection threshold (MDT) and vibration detection threshold representing the function of large myelinated fibers or central pathways, cold detection threshold (CDT), cold pain threshold, warm detection threshold (WDT), thermal sensory limen (TSL), heat pain threshold (HPT), presence of paradoxical heat sensations, mechanical pain threshold, mechanical pain sensitivity (MPS), and pressure pain threshold representing small fiber function or central pathways. In addition, the presence of dynamic mechanical allodynia (DMA) and temporal summation of pain (wind-up ratio) are assessed. Parameters were tested as described previously.^11^ Patients were tested bilateral on the affected and corresponding contralateral body side. Testing always started on the unaffected, ie, corresponding contralateral side before the affected area of maximal pain (test area) was tested.

### 2.3. Statistical analysis

Quantitative sensory testing results were analyzed according to published guidelines^29^ and compared with a reference database of healthy controls.^20^ To make the patients' data comparable with the values of healthy controls, individual patient data were normalized to the respective sex, age group, and tested area of the healthy controls usin*g z*-values (*z* = [individual value − mean database]/SD database). Resulting *Z* scores above “0” indicate hyperfunction, ie, patients are more sensitive to the tested parameter compared with controls (lower thresholds), whereas *Z* scores below “0” indicate hypofunction and therefore a loss of or lower sensitivity of the patient compared with controls (higher thresholds). Because PHN is a unilateral disease, both *z*-values out of the 95% confidence interval (absolute abnormal value) in the affected area and a difference of more than 2 SDs in the *z* scores between affected and corresponding contralateral area (abnormal side-to-side difference) were considered as abnormal values. The Wilcoxon test was used for intragroup comparison, ie, analysis between affected and contralateral side as well as between baseline and follow-up within the groups. Linear relationships were assessed by Pearson correlation coefficient. Frequencies of abnormal QST values were analyzed with the χ^2^ test. *P* < 0.05 was considered statistically significant.

## 3. Results

### 3.1. Somatosensory findings at baseline of the whole cohort

On baseline, patients had higher frequencies for loss of thermal (cold detection: n = 11, *P* < 0.001; warm detection: n = 14, *P* < 0.001) and mechanical detection (n = 14, *P* < 0.001; vibration detection: n = 3, *P* < 0.001) as well as heat pain (n = 3, *P* < 0.001) in the affected area compared with the reference values of healthy controls. Mechanical pain sensitivity to pinprick stimuli was either increased (MPS: n = 4, *P* < 0.001) or decreased (mechanical pain threshold: n = 6, *P* < 0.001; MPS: n = 4, *P* < 0.001), whereas pain sensitivity to blunt pressure was increased (n = 7, *P* < 0.001) compared with healthy controls. Dynamic mechanical allodynia and paradoxical heat sensitivity were present in 40% and 20%, respectively. None of the QST parameters at baseline correlated with pain intensity or disease duration, but z-values for thermal thresholds were lower, the older the patients were (CDT: *R* = −0.61, *P* < 0.01; WDT: −0.51, *P* < 0.05).

Based on frequencies of abnormal QST values, 2 subgroups could be distinguished: 14 (70%) PHN patients presented with impaired C-fiber function, ie, loss of warm detection. Six (30%) patients had preserved C-fiber function, ie, no abnormalities in warm detection or HPTs, whereas one patient additionally demonstrated sensitized C-fiber function, ie, no abnormalities in warm detection, but increased heat pain sensitivity. Patients with impaired C-fiber function did not differ from patients with preserved C-fiber function regarding age, disease duration, or pain intensity at baseline (Table 1).

**Table 1:**
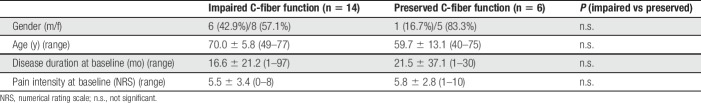
Characteristics of subgroups at baseline.

### 3.2. Comparison of patients with impaired and preserved C-fiber function at baseline

Quantitative sensory testing values of the 2 subgroups are shown in Table 2.

**Table 2:**
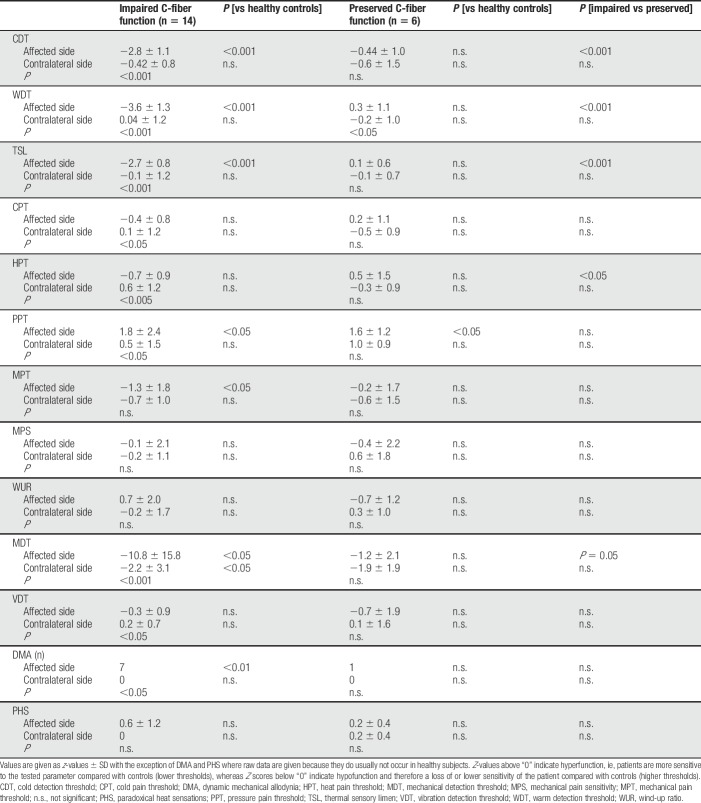
Quantitative sensory testing values of subgroups at baseline.

Patients with impaired C-fiber function had loss of thermal (CDT, WDT, and TSL) and mechanical (MDT and vibration detection threshold) detection as well as decreased sensitivity to thermal pain (cold pain threshold and HPT), but increased sensitivity to blunt pressure and DMA on the affected side compared with the contralateral side (Table 2). All patients had loss of detection: 12 patients (85.7%) had a combined loss of detection (loss of small and large fiber function) and 2 presented with a solely loss of small fiber function. This loss of detection was combined with increased pain sensitivity in 9/14 (64.2%) patients with either solely increased mechanical (n = 8) or mixed (thermal and mechanical, n = 1) gain of function.

By contrast, patients with preserved C-fiber function did not differ between the affected and contralateral side regarding gain or loss of thermal or mechanical detection or pain thresholds (Table 2). The only exception here was that z-value for WDT was higher on the affected compared with the contralateral side meaning that sensitivity for warm detection was increased on the affected compared with the contralateral side representing hypersensitivity (Table 2). Despite preserved C-fiber function, 2 patients (33.3%) presented with loss of mechanical detection, ie, a dysfunction of A-beta fibers or central pathways, which was not combined with increased pain sensitivity. Three of the 4 patients without loss of detection had abnormal increased sensitivity to mechanical (DMA, n = 1), painful cold (n = 1), or painful heat and blunt pressure stimuli (n = 1).

Patients with impaired C-fiber function had a stronger loss of detection for thermal (CDT, WDT, and TSL) and mechanical (MDT) detection and painful heat on the affected side compared to patients with preserved C-fiber function (Table 2).

### 3.3. Change of pain intensity and medication in the course of the disease

Mean pain intensity did not change between baseline and follow-up measurement, neither in patients with impaired (5.3 ± 1.1 vs 5.2 ± 1.1, *P* = not significant [n.s.]) nor with preserved C-fiber function (4.2 ± 2.8 vs 5.8 ± 2.8, *P* = n.s.). Only 4/14 patients (28%) improved in pain intensity from baseline to follow-up in the subgroup with impaired and 3/6 (50%) patients with preserved C-fiber function (*P* = n.s.). Between baseline and follow-up, pain medication was unchanged in 2 patients (10%; n = 1 in the impaired and n = 1 in the preserved subgroup), whereas the number of coanalgesics was reduced in 7 (35%; n = 6 [42.9%] in the impaired and n = 1 [16.6%] in the preserved subgroup) and increased in 11 patients (55%; n = 7 [50%] in the impaired and n = 4 [66.7%] in the preserved subgroup). Interestingly, change in pain intensity did not differ between those patients where the number of pain medication was increased or decreased (−1.1 ± 2.2 vs −0.7 ± 3.0, *P* = n.s.).

### 3.4. Change of somatosensory function in patients with impaired C-fiber function in the course of the disease

**Figure 1.**
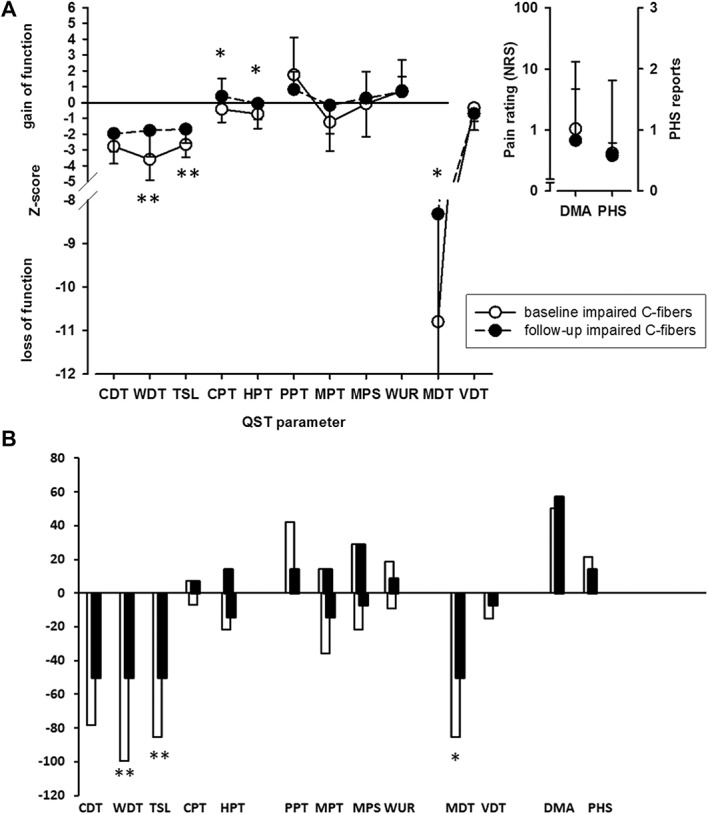
Somatosensory profile and the presence of dynamic mechanical allodynia and paradoxical heat sensations (A) as well as frequencies of abnormal values (B) on baseline (white circles and white columns) and follow-up (black circles and black columns) examination in PHN patients with impaired C-fiber function. CDT, cold detection threshold; CPT, cold pain threshold; DMA, dynamic mechanical allodynia; HPT, heat pain threshold; MDT, mechanical detection threshold, MPS, mechanical pain sensitivity; MPT, mechanical pain threshold; NRS, numerical rating scale; PHN, postherpetic neuralgia; PHS, paradoxical heat sensitivity; PPT, pressure pain threshold; QST, quantitative sensory testing; TSL, thermal sensory limen; VDT, vibration detection threshold; WDT, warm detection threshold; WUR, wind-up ratio. *P < 0.05 and **P < 0.01 for comparison of baseline vs follow-up measurement.

Patients with impaired C-fiber function at baseline showed an improvement of warm and mechanical (MDT) detection as well as an increase in cold and heat pain sensitivity in the follow-up examination (Fig. 1A). Change in pain intensity did not correlate with change in QST parameters.

Changes in the frequencies of abnormal values are shown in Figure 1B. On the individual level, 7 patients showed a normalization of warm and 5 of MDTs (Table 3). Overall, all detection thresholds showed a trend to normalization, whereas pain sensitivity increased or decreased on follow-up without a clear trend (Fig. 1B), ie, there was neither a decreased pain sensitivity nor a sensitization of the nociceptive system.

**Table 3:**
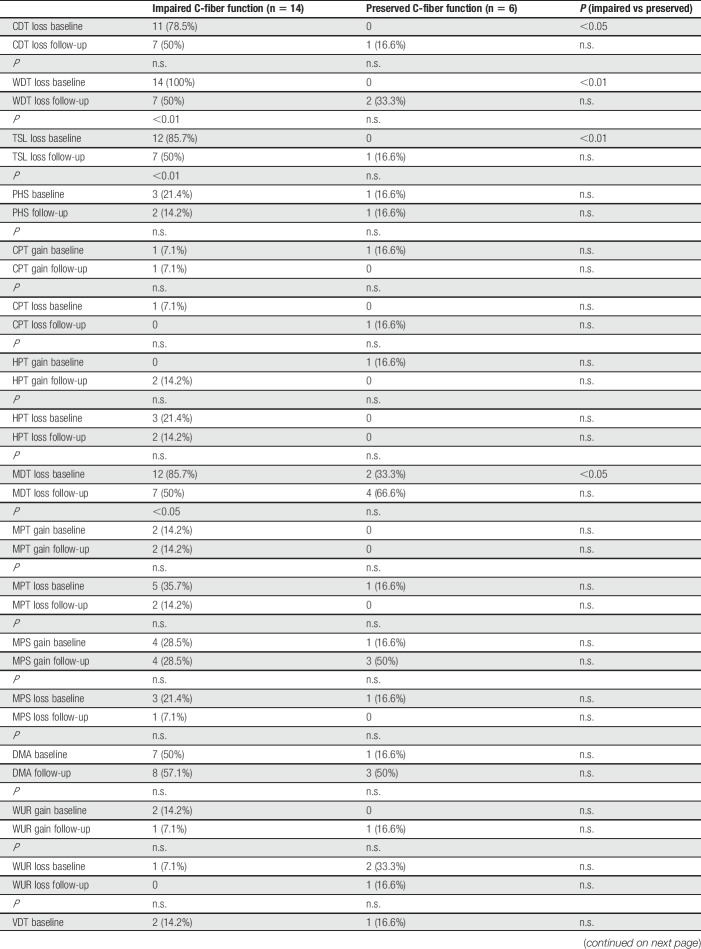

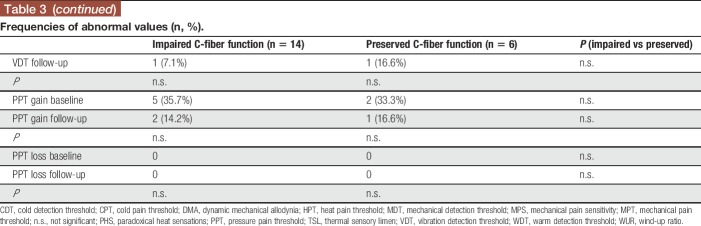
Frequencies of abnormal values (n, %).

No differences were observed between patients with short or long disease duration. Interestingly, patients with a lower pain intensity (NRS ≤6, n = 5, mean 1.6 ± 2.3 NRS) had a *stronger* loss of mechanical detection on baseline (−24.9 ± 20.3 vs −3.0 ± 1.5, *P* < 0.05) as well as mechanical detection (−20.5 ± 23.9 vs −1.5 ± 1.1, *P* < 0.005) and TSL (−2.3 ± 0.5 vs −1.4 ± 0.9, *P* < 0.05) on follow-up testing compared to those with a high pain intensity (n = 9 ≥ 7 NRS; 7.7 ± 1.0). No differences of QST parameters at baseline or follow-up were observed between those with and without a reduction of pain intensity between baseline and follow-up examination.

### 3.5. Change of somatosensory function in patients with preserved C-fiber function in the course of the disease

**Figure 2.**
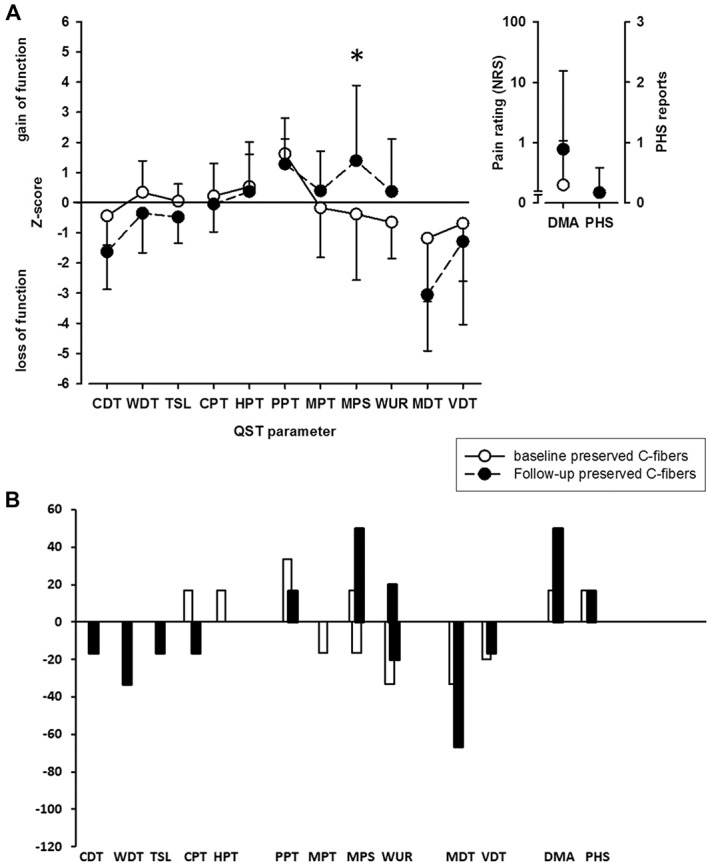
Somatosensory profile and the presence of dynamic mechanical allodynia and paradoxical heat sensations (A) as well as frequencies of abnormal values (B) on baseline (white circles and white columns) and follow-up (black circles and black columns) examination in PHN patients with preserved C-fiber function. CDT, cold detection threshold; CPT, cold pain threshold; DMA, dynamic mechanical allodynia; HPT, heat pain threshold; MDT, mechanical detection threshold; MPS, mechanical pain sensitivity; MPT, mechanical pain threshold; NRS, numerical rating scale; PHN, postherpetic neuralgia; PHS, paradoxical heat sensitivity; PPT, pressure pain threshold; QST, quantitative sensory testing; TSL, thermal sensory limen; VDT, vibration detection threshold; WDT, warm detection threshold; WUR, wind-up ratio. *P < 0.05 for comparison of baseline vs follow-up measurement.

In contrast to patients with impaired C-fiber function, patients showed an increase in MPS on follow-up (Fig. 2A). Furthermore, patients showed a trend towards a stronger loss of detection. This trend could also be observed on the individual level (Table 3 and Fig. 2B). The highest changes of frequencies between baseline and follow-up were observed for MDT (loss in 2 more patients on follow-up) and MPS (gain in 2 more patients and DMA: in 2 more patients, Table 3). Difference in pain intensity did not correlate with change in QST parameters.

No differences were observed between patients with short or long disease duration or between those with higher (≥7 NRS, n = 4) or lower (n = 2) pain intensity within the group of patients with preserved C-fiber function.

### 3.6. Comparison of change of somatosensory function of preserved and impaired C-fiber function in the course of the disease

Patients with impaired C-fiber function did not differ from patients with preserved C-fiber function in change in pain intensity (−0.5 ± 2.3 vs −1.7 ± 2.6 NRS, *P* = n.s.), pain intensity at follow-up examination (5.0 ± 3.9 vs 4.2 ± 2.8 NRS, *P* = n.s.), time in between the 2 examinations (26.9 ± 19.6 vs 42.7 ± 30.8 months, *P* = n.s.), or disease duration (43.5 ± 3.8 vs 64.2 ± 63 months, *P* = n.s.) at follow-up. Patients with impaired C-fiber function improved in thermal and MDTs as well as DMA compared to those with preserved C-fiber function (CDT: 0.8 ± 1.4 vs −1.2 ± 1.2, WDT: 1.8 ± 2.1 vs −0.7 ± 1.7; TSL: 1.0 ± 1.1 vs −0.5 ± 1.3; MDT: 2.5 ± 7.8 vs −1.9 ± 3.1, DMA: −5.6 ± 12.2 vs 11.4 ± 17.8, *P* < 0.05 for all, Figs. 1A and 2A).

## 4. Discussion

This study shows that in the course of PHN, (1) pain reduction is independent from the subtype of PHN and (2) patients with impaired and preserved C-fiber function differ regarding change of somatosensory function: Although patients with impaired C-fiber function at baseline improve in loss of thermal and mechanical detection independent from the improvement in pain intensity and even after a long duration of disease, ie, show a shift towards a normalization of the somatosensory profile, patients with preserved/sensitized C-fiber function are more predisposed to develop signs of central sensitization, ie, an increase in MPS. Results therefore suggest that C-fiber function is able to improve even after a long time, as the disease duration at baseline measurement was already more than 1 year after disease onset and follow-up examination about 3 years later. This is in line with the observation of another follow-up examination of patients with herpes zoster that demonstrated an improvement of sensory thresholds 3.9 to 7.7 years after herpes zoster, despite the presence of somatosensory abnormalities and partly missing reinnervation of herpes zoster affected on skin biopsy.^27^

It is unlikely that a functional loss, ie, pain-induced hypoaesthesia^8^ plays a major role because pain intensity did not change in this subgroup, change in somatosensory profiles was independent from improvement in pain intensity and patients with a lower pain intensity demonstrated a stronger loss on QST.

The findings of an increasing (central) sensitization of the nociceptive system in the preserved/sensitized subtype of PHN are in line with current concepts of pain chronification demonstrating that central sensitization can develop as a result of ongoing C-fiber activity of intact C fibers.^2^ In addition, in this group, there was a trend towards a development of a mechanical and thermal loss of detection. Of course, it has to be kept in mind that this subgroup of patients within the study was small; however, with regards to the increasing central sensitization, this progressive loss could be secondary to nociceptive stimulation^8^ rather than due to degeneration of peripheral nerve fibers. Although the change in somatosensory profiles was independent from improvement in pain intensity, central sensitization of the nociceptive system was clearly visible on the somatosensory profile. Processes underlying central sensitization (for review, see Ref. 34) might also be able to induce secondary hypoesthesia, as it has been observed in experimental and chronic pain.^8,18,19,24,36^

Results of the change of the somatosensory phenotype within the course of PHN further support the view that the pain profile is dynamic, ie, can change within the same individual as a consequence of individual characteristics such as age, sex, genetic phenotype, prior medical history including primary and secondary psychological factors,^13,25,33^ as well as painful events and treatment.^35^ Regarding the idea of a mechanism-based treatment in neuropathic pain^9,10,32^ based on the somatosensory phenotype,^1^ a change of the somatosensory phenotype within the course of a disease would have major influence on treatment. For example, patients with a strong loss of detection due to degeneration might respond better to treatment with anticonvulsants or topical agents when regeneration occurs.^5,6,22^ On the other hand, in the sensitized/preserved phenotype, it might be more important to prevent further mechanisms of pain chronification, ie, by a more aggressive analgesic treatment.^3,4,12,17,23^

One limitation of this study is of course the small study number, especially in the preserved/sensitized subtype of PHN. Thus, the conclusions that can be drawn from this subgroup are limited. In addition, treatment was not stable in the patients; however, because mean pain intensity did not change between baseline and follow-up and change in pain intensity did not differ between those patients where the number of pain medication was increased or decreased, this should not have major influence on our results.

In conclusion, our results suggest a dynamic of the somatosensory profile and that follow-up examinations of the somatosensory phenotype to assess underlying mechanisms are important to optimize treatment.

## Disclosures

J. Gierthmühlen has received speaker fees and travel support from Pfizer, Grünenthal, Sanofi Pasteur MSD GmbH, TAD Pharma and consultancy fees from Glenmark. A. Binder received honoraria from Astellas, Pfizer, Allergan, Grünenthal, Boehringer Ingelheim, Bayer, and advisory from Grünenthal, Genzyme, Pfizer, Boehringer Ingelheim, and Bayer. S. Rehm reports travel support from Grünenthal and speaker fees from Bayer Vital. R. Baron has received grants/research support from Pfizer, Genzyme, Grünenthal, and Mundipharma. He is a member of the EU Project No 633491: DOLORisk. A member of the IMI “Europain” collaboration and industry members of this are: AstraZeneca, Pfizer, Esteve, UCBPharma, Sanofi Aventis, Grünenthal, Eli Lilly, and Boehringer Ingelheim. German Federal Ministry of Education and Research (BMBF): Member of the ERANET NEURON/IM-PAIN Project. German Research Network on Neuropathic Pain, NoPain system biology, and German Research Foundation (DFG). He has received speaking fees from Pfizer, Genzyme, Grünenthal, Mundipharma, Sanofi Pasteur, Medtronic, Eisai, Lilly, Boehringer Ingelheim, Astellas, Desitin, Teva Pharma, Bayer-Schering, MSD, and bioCSL. He has been a consultant for Pfizer, Genzyme, Grünenthal, Mundipharma, Allergan, Sanofi Pasteur, Medtronic, Eisai, Lilly, Boehringer Ingelheim, Astellas, Novartis, Bristol-Myers-Squibb, Biogenidec, AstraZeneca, Merck, Abbvie, Daiichi Sankyo, Glenmark Pharmaceuticals, Genentech, and bioCSL. The remaining authors have no conflict of interest to declare.

This work was supported by Pfizer Germany. The sponsor was not involved in design and conduct of the study, in collection, management, analysis, interpretation, and preparation of the data, and in review or approval of the manuscript.
